# Supplementary material for: Over 13,000 elements from a single bonebed help elucidate disarticulation and transport of an Edmontosaurus thanatocoenosis
Source: PLoS One. 2020 May 21;15(5):e0233182. doi: 10.1371/journal.pone.0233182 (PMC7241792; doi:10.1371/journal.pone.0233182)
Supplement: Attachment — Submitted filename: Snyder et al 2020_manuscript_final-REVIEWED.docx [file pone.0233182.s001.docx]

**Over 13,000 elements from a single bonebed help elucidate disarticulation and transport of an *Edmontosaurus* thanatocoenosis.**

Taphonomy of an *Edmontosaurus*bonebed.

Keith Snyder^1^, Matthew McLain^2^, Jared Wood^3*^, Arthur Chadwick^3^

^1^Biology Department, Southern Adventist University, Collegedale, TN, USA

^2^Biological and Physical Sciences, The Master’s University, Santa Clarita, CA, USA

^3^Department of Biological Sciences, Southwestern Adventist University, Keene, TX, USA

*Corresponding author

E-mail: j.wood@swau.edu

**Abstract**

Over twenty years of work on the Hanson Ranch (HR) Bonebed in the Lance Formation of eastern Wyoming has yielded over 13,000 individual elements primarily of the hadrosaurid dinosaur *Edmontosaurus annectens*. The fossil bones are found normally-graded within a fine-grained (claystone to siltstone) bed that varies from one to two meters in thickness. Almost all specimens possess exquisite preservation (i.e., little to no abrasion, weathering, and breakage), but they are disarticulated which, combined with our sedimentological observations, suggests that the bones were remobilized and buried after a period of initial decay and disarticulation of *Edmontosaurus* carcasses. Because of the large number of recovered fossil elements, we have been able to gain deeper insight into *Edmontosaurus* taphonomy, including disarticulation and transport of skeletal elements. The most common postcranial elements in the bonebed were pubes, ischia, scapulae, ribs, and limb bones. By contrast, vertebrae, ilia, and chevrons were rare. The most common craniomandibular bones included dentaries, nasals, quadrates, and jugals, whereas the premaxillae, predentaries, and braincase bones were underrepresented. Thus, we see a common pattern that the axial-most bones in the skeleton (cranial and postcranial) as well as the distal-most elements (e.g., predentary, premaxilla, phalanges, etc.) were the most underrepresented. We hypothesize that following decay and disarticulation, hydraulic winnowing removed the articulated sections (e.g., vertebral columns) and the small, distal-most elements before the remaining bones were swept up in a subaqueous debris flow that generated the deposit. Comparison of the HR Bonebed with other widely dispersed Upper Cretaceous hadrosaurid-dominated bonebeds reveals many shared attributes, which suggests similar processes at work in the formation of these bonebeds across space and time.

**Introduction**

*Edmontosaurus*-dominated bonebeds are widespread in the Upper Cretaceous (Campanian – Maastrichtian) strata of western North America and have been reported recently from the Dinosaur Park [1], Horseshoe Canyon [2 - 3], Two Medicine [4], Hell Creek [5], Prince Creek [6], and Lance Formations [7 - 8].

An upper Maastrichtian *Edmontosaurus annectens* monodominant bonebed of the Hell Creek Formation has recently been described by Ullmann et al. [9], with comparisons to other similar published bonebeds. By examining the host matrix, ontogenic stages, probable cause of death, state of articulation of the remains, and various bone surface modifications, a broader picture is emerging of the paleobiology and paleoecology of this species.

Limited information for the Lance Formation (upper Maastrichtian) has been presented in an unpublished thesis by Christians [10] and this Formation has been mentioned tangentially by Colson et al. (5) in a stratigraphic publication. Sternberg [11], Tedesco et al. [12], Chadwick et al. [8], and Weeks [13] have identified other *E. annectens* bonebeds in the Lance Formation, but none have been described in detail in the scientific literature.

This study examines in detail the taphonomy and depositional history of an extensive *E. annectens* bonebed in the Lance Formation of eastern Wyoming. The Hanson Ranch (HR) Bonebed includes five main quarries and three exploratory quarries. Approximately 13,000 elements (including ~8,400 identifiable bones) have been recovered in 506 m^2^ of excavated area between 1996 and 2016. All fossils that have been collected are housed at Southwestern Adventist University (SWAU) in Keene, TX, and can be examined online at <http://fossil.swau.edu>. Through the description of the taphonomy of this site, and comparisons of this site with other Upper Cretaceous hadrosaurid bonebeds, we hope to gain insight into the complexity of catastrophic death assemblages and the paleobiology of *E. annectens*.

**Geological Setting**

The Lance Formation of northeastern Wyoming is the uppermost deposit of the Cretaceous in the Powder River Basin. It is overlain by the Paleocene Fort Union Formation and rests upon the Fox Hills Formation. The contact with the Fox Hills Formation is conformable and is often difficult to identify. Upper Fox Hills Formation sediments probably interfinger with those of the lowermost Lance Formation in most outcrops [14]. Combined with the underlying Fox Hills Formation sandstone, the Lance Formation attains thicknesses in excess of 1 km in the southern part of the basin, as determined from well cores [14].

During deposition of the Lance, the region presently represented by the Powder River Basin was located on the western margin of the Western Interior Seaway. The southward thickening of the Lance and easterly directed paleocurrents [13 - 15] indicate that the tectonics responsible for forming the basin had not yet influenced sedimentation and probably did not do so in this region until well after the Cretaceous-Paleogene (K-Pg) boundary [16]. Dickenson et al. [17] recognized that marine foreland sedimentation persisted in the Powder River Basin longer than in other peripheral Laramide basins, and that in this region marine influence continued well into the Paleocene.

The Lance sediments accumulated on top of marine and marginal marine floodplains in an area of low relief [14, 18]. Paleocurrents and possible source areas for the Lance sediments indicate that the clastic materials were derived from the west, probably from west of the present Bighorn Basin [14 – 15, 17]. Sediments in the study area are poorly indurated, dark gray to black claystones and mudstones that weather to light gray on exposed surfaces, and tan to white, fine to medium-grained immature lithic sandstones that include granitic rock fragments. Connor [14] suggested sedimentary cover of the emerging Washakie, Owl Creek, and Wind River mountains as a possible source of these sediments. Detritus derived from the emerging Granite Mountains [19] may have contributed the granitic rock fragments.

The *Edmontosaurus* bonebed that is the subject of this paper is located in the eastern limb of the Powder River syncline in north-central Niobrara County, WY (Fig 1). The bonebed occurs in the middle to upper part of the Upper Cretaceous (Maastrichtian) Lance Formation. Because of the low angle of dip (1-3 degrees to the west), the thickness of the unit, and the scarcity of identifiable stratigraphic marker beds, the position of the bonebed within the Lance was estimated geometrically [20].

**Fig 1. Hanson Ranch Bonebed site on the Lance Formation Map.**

**Sedimentology**

The Lance Formation, in the study area and generally throughout its exposed extent, consists of poorly defined strata of claystone, mudstone, and immature, fine-grained quartz and lithic sandstone. Because cementation is generally absent or is secondary calcite, much of the exposure of the Lance manifests itself as “badlands topography.” Occasional beds of white sandstone are up to 5 meters thick. Rapid deposition of the sandstone is attested to by beds displaying seismic disruptions that do not extend into the underlying or overlying sediments (Fig 2), indicating that dewatering had not occurred prior to the seismic event [13].

**Fig 2.  Sandstone unit showing effects of seismic modification of primary sedimentary structures. Note that the underlying beds are not affected. The capping rock is also sandstone, but it is diagenetically cemented with carbonate. Hammer bottom left for scale.**

Lance stratigraphy is problematic since marker beds are very difficult to find. The most prominent “beds” are typically poorly cemented sandstones that have secondarily been cemented with calcite. Such cementation may persist for many meters or may disappear in just a few meters. Even a persistent bed that can be traced for 100 meters or more, may change character completely in only 20 meters, laterally grading into fine-grained sandstones, mudstone, or even claystones. Because of this lack of persistent bedding, placing quarries in the Lance Formation in a stratigraphic framework is exceedingly difficult. Often the bones themselves provide the only reliable stratigraphic marker. Recently, a disturbed sandstone bed has been mapped over an area of approximately 50 km^2^ [13]. Using this bed as a chronostratigraphic horizon, we have been able to place the quarries into approximate stratigraphic position (Fig 3).

**Fig 3. Local stratigraphy of the main bonebeds at the Hanson Research Station.**

The bonebed in the main quarry area is overlain by a bed of tan, poorly cemented, fine-grained, immature quartz sandstone in conformable contact with the underlying mudstone. The sediment containing the bones is a one- to two-meter-thick, light to medium gray (sometimes almost black in fresh exposures), claystone to siltstone, which is also poorly to moderately indurated. Weathered surfaces, when wet, quickly revert to mud. The bones are found within the claystone, generally in the lower part, in a normally graded bed. The largest bones may rest on the bottom of the bed, or where the bed is thicker, bones appear to be entirely suspended within the mudstone. The underlying bed is often a layer of greenish, moderately well-cemented quartz sandstone with secondary chlorite cement. This layer, generally only a few centimeters thick, may rest on an orange-weathering, well-cemented quartz sandstone with carbonate cement.

**Methods and Materials**

**Specimen Preparation and Taphonomic Classifications**

The HR *Edmontosaurus* Bonebed was evaluated from 1995 – 1999 with the removal of 135 elements. Concentrated work began in 2000 and the bed has been worked each summer since. The data in this paper extend to the 2017 season. All specimens have been recorded except for small *Edmontosaurus* teeth less than 1 cm, short ossified tendons less than 5 cm, and unrecognizable fragments less than 1 cm in greatest length.

The quarries were initially opened where bones were exposed at the surface. Elements were excavated using standard techniques [21]. Field techniques using pre-printed cards, digital photographs of specimens *in situ*, descriptive drawings and measurements in a field book, and high-precision RTK GPS positioning were followed as described in Chadwick et al. [22]. Data were entered into ArcGIS to create three-dimensional maps of the quarries and bones [23].

Cleaning and stabilization were completed in the laboratory. All elements were then re-photographed and their data reconfirmed and entered into the online database. Each element was photographed in 360° to allow virtual examination via the internet (see http://fossil.swau.edu). Standard taphonomic procedures were followed as developed by Behrensmeyer [24], enhanced by Ryan et al. [25] and Eberth et al. [26], and advocated for comparative purposes between bonebeds by Lyman [27] and Moore [28]. Data were collected for fracturing, abrasion, weathering, pathologies, surface markings, crushing, sizes, and percentage completeness. These data and information on taxonomy, anatomy, and spatial position in the quarries were used to complete each specimen profile.

We have employed the suggestions of Ryan et al. [25] as a basis for the four stages of weathering as it adds slightly to Fiorillo’s [29] simplification of Behrensmeyer’s [30] original publication. These stages are: Stage 0 = Fossil surface shows no sign of cracking or flaking due to weathering, Stage 1 = Surface cracking parallel or subparallel to fiber structure, Stage 2 = Cracks starting to penetrate into the marrow cavity and surface flaking away from cracks, Stage 3 = Large chunks of outer laminated bone have flaked away.

The four abrasion categories classified by Ryan et al. [25] were also used. These stages are: Stage 0 = Pristine and unabraded surface, Stage 1 = Broken edges are rounded, Stage 2 = All edges are well-rounded, and Stage 3 = All edges extremely rounded, processes appear as bone protrusions only. The three major types of bone fracturing used for classification (longitudinal, transverse, and spiral/greenstick) were originally from Haynes [31] and subsequently clarified by Ryan et al. [25]. We have added “indeterminate” as an additional classification for those not readily fitting into these categories.

Element orientation was obtained from the database, by isolating 268 femora, tibiae, fibulae, humeri, radii, and fibulae from the four largest quarries. Orientation was analyzed in R (version 3.6.0) using *CircStats* (version 0.2-6) and *Circular* (version 0.4-93) packages. Rose diagrams were also generated in R using the *ggplot2* package (version 3.1.1). Spearman’s correlation analyses were done using R and the *stats* package (version 3.6.0).

Due to the continued challenges of using Voorhies groupings for element transport and distribution, we followed the suggestion of Gangloff and Fiorillo [6] to instead consider the number of individual elements from the HR Bonebed to give a better understanding of the possible survivability and distribution of elements.

Approximately 11,000 citizen scientist working days were recorded, enabling public participation by well over a thousand people over 20 years of excavation.

**Species Identification**

In the northwestern U.S. and southwestern Canada, two species of *Edmontosaurus* are recognized: *E. regalis* (naming citation) and *E. annectens* (naming citation). The main quarries expose a monodominant bonebed of this genus. Campione and Evans [32] assert that *E. regalis* are found in the upper Campanian and *E. annectens* are found in the upper Maastrichtian. *E. regalis* appears to be limited to the upper Campanian and lower Maastrichtian [33]. Ullmann et al. [9] agree and have built a strong case for Hell Creek material belonging to *E. annectens*. This species is found in the Lance, Hell Creek, Laramie, Scholard, and Frenchman formations of Colorado, Wyoming, South Dakota, Montana and Alberta, all of which pertain to the upper Maastrichtian [34]. The HR bonebed is located in the southern portion of the range of the Lance Formation, leading us to conclude all hadrosaurine elements are of the species *E. annectens*.

**Results**

**Attributes of Bonebed**

The HR Bonebed is exposed along the eastern margin of a local transition from pasturelands in the east to broken topography of canyons and ridges in the west, referred to locally as “The Breaks.” This erosional relief has exposed an extensive layer of bones along the sides of four successive canyons arising from a single ridge. This bonebed is the subject of much of our research and at present is the target of our five main quarries: North, South, Southeast, Teague, and West in addition to three exploratory quarries: Toe, Neufeld, and DKC (Fig 4). Erosion has removed the main bonebed on the west, south, and southeast sides, limiting the ability to measure the original extent of the bonebed. The exploratory quarries are beginning to help define the northern and northeastern boundaries of this apparently contiguous layer. Areas between the quarries have as much as seven meters of overburden, limiting access in places.

**Fig 4. Three dimensional quarry map. White dots indicate location of individual bones, outlining the bonebed layer through the hills between formal excavation sites. The black dots indicate where bones were collected in the quarries.**

Typically, the bones are found in mudstone to claystone, which is easily removed from the specimen. However, some bones are partially or entirely encased in carbonate-cemented mudstone that is very hard and generally leaves the bones affected by the removal process. The formation of a carbonate case is thought to result from the release of organic constituents, particularly fatty acids, to the surrounding matrix. This and the production of ammonia from protein degradation probably caused the precipitation of carbonate in the matrix [35 - 36]. Such a process is consistent with the burial of bones occurring while they were still relatively fresh.

**Bone Density**

Our five main quarries, examining the thickest portion of the bonebed, include North (N), South (S), Southeast (SE), West (W), and Teague (T). Three exploratory quarries were also opened to determine the lateral extent of the bonebed; these include Toe, Neufeld (Neu), and DKC. The total excavation of 508 m^2^ has yielded over 13,000 specimens including identifiable bones, teeth, ossified tendons, and bone fragments (Fig 4, Table 1). Screen washing was implemented in several of the quarries, and those data are included in Table 2.

**Table 1. Bone densities at the five main and three exploratory quarries. Abbreviations:…**

| Quarries | N | S | SE | T | W | Toe | Neu | DKC | Total |
| --- | --- | --- | --- | --- | --- | --- | --- | --- | --- |
| Square meters excavated | 190 | 129 | 78 | 45 | 20 | 20 | 20 | 6 | 508 |
| Total bones | 5385 | 3858 | 2318 | 1053 | 554 | 123 | 304 | 30 | 13,625 |
| Bone density B/m^2^ | 28.3 | 29.9 | 29.7 | 23.4 | 28 | 6.2 | 15.2 | 5 | 26.8 |

**Table 2. HR Bonebed taxa represented.**

| Mollusca |  |  |  |  |  |
| --- | --- | --- | --- | --- | --- |
|  | Gastropoda indet. |  |  |  | 27 |
|  | Pelecypoda (Bivalvia) indet. |  |  |  | 16 |
| Chondrichthyes |  |  |  |  |  |
|  | Rajiformes – Rajidae (skates) |  |  |  | 3 |
| Actinopterygii |  |  |  |  |  |
|  | Lepisosteiformes – Lepisosteidae – *Lepisosteus* (gar) |  |  |  | 5 |
|  | Actinopterygii indet. |  |  |  | 18 |
|  |  | Teleostei indet. |  |  | 2 |
| Reptilia |  |  |  |  |  |
|  | Reptilia indet. |  |  |  | 165 |
|  | Testudines indet. (turtles) |  |  |  | 74 |
|  | Squamata indet. (lizards and snakes) |  |  |  | 4 |
|  | Archosauria |  |  |  |  |
|  |  | Aves indet. (birds) |  |  | 30 |
|  |  | Crocodylia indet. |  |  | 34 |
|  |  |  | Crocodyloidea - *Leidyosuchus* |  | 17 |
|  |  |  | Alligatoroidea - *Brachychampsa* |  | 34 |
|  |  | Saurischia |  |  |  |
|  |  |  | Theropoda – indet. |  | 121 |
|  |  |  | Tyrannosauridae |  |  |
|  |  |  |  | *Tyrannosaurus* | 56 |
|  |  |  |  | *Nanotyrannus* | 135 |
|  |  |  |  | *Aublysodon* – like forms | 3 |
|  |  |  | Troodontidae - *Troodon* |  | 52 |
|  |  |  | Dromaeosauridae |  |  |
|  |  |  |  | *Acheroraptor* | 128 |
|  |  |  |  | *Dromaeosaurus* indet. | 3 |
|  |  |  | Coelurosauria indet. - *Richardoestesia* |  | 5 |
|  |  |  | Ornithomimosauria - *Struthiomimus* |  | 4 |
|  |  | Ornithischia |  |  |  |
|  |  |  | Hadrosauridae - *Edmontosaurus* |  | 12,503 |
|  |  |  | Pachycephalosauridae – *Pachycephalosaurus* |  | 5 |
|  |  |  | Ceratopsidae - *Triceratops* |  | 103 |
|  |  |  | Nodosauridae - *Nodosaurus* |  | 4 |
|  |  |  | Thescelosauridae - *Thescelosaurus* |  | 4 |
| Mammalia indet. |  |  |  |  | 2 |

Table 1 summarizes the square meters excavated, the total bones removed, and the bone density per square meter in the eight quarries. Although small by location, thickness of the bone layer, and skeletal element density, West quarry is part of the larger main quarries. Density numbers of approximately 23 –30 elements per square meter (e/m^2^) are similar to those reported for a *Centrosaurus* bonebed in the Dinosaur Park Formation [25], suggesting that these two beds may share a similar taphonomic history. The three exploratory quarries (Toe, Neufeld, DKC) have fewer elements per square meter, but vary in element concentration. Although Neufeld quarry (15 e/m^2^) is farther away from the main quarries than either Toe quarry (6.15 e/m^2^) or DKC quarry (5 e/m^2^), it has a higher concentration of elements per square meter. Further quarrying may elucidate the reason for this difference.

A representative quarry map for North quarry is shown in Fig 5. The thicknesses of the HR bonebed in the main quarries of 90-140 cm and 30-50 cm in the exploratory quarries fall within recently reported dinosaur bonebed thicknesses from other Cretaceous sites across North America: 35–90 cm [6], 40–60 cm [37], 40–150 cm [38], and maximum thicknesses for three sites of 60 cm, 120 cm, and 150 cm [3]. That the bonebed horizon is thicker in the main quarries and thinner in the outer, exploratory quarries is consistent with a lenticular geometry for this layer.

**Fig 5. Plan map of the elements excavated in a representative quarry (North). Marker dots are spaced in meter increments.**

Of the total elements identified in the quarries (13,625), approximately 14.7% are unidentifiable bone fragments, 6.7% are *Edmontosaurus* teeth, and 17.2% are tendons (Fig 6). We have removed these from further discussion since they offer little additional information pertinent to this paper.

**Fig 6. Skeletal element distribution for main quarries. Percentages out of 12,303 *Edmontosaurus* elements only.**

**Articulation**

In the HR Bonebed, skeletal elements are completely disarticulated with three exceptions. In North quarry, three complete *Edmontosaurus* caudal vertebrae were found articulated. A femur, tibia, fibula, calcaneum and astragalus were also found articulated. The only other associated bones were two *Edmontosaurus* tibiae found adjacent to each other in South quarry that appeared to be a paired set. Thus <0.1% of the bones found show actual or possible articulation.

**Vertical Distribution**

All quarries exhibit fairly uniform normal grading of the bones, with smaller bones at the top and larger bones at the bottom. The first bones encountered are consistently unguals or phalanges, then smaller ribs, vertebrae, indeterminate bone fragments and chevrons. Large bones such as tibiae, fibulae, and femora and metatarsals are consistently found at the very bottom (Fig 7). Southeast and Teague quarries are more condensed in the lowest layer, resulting in many bones in contact. Ossified tendons and teeth are found randomly throughout the bonebed.

**Fig 7. Plan view of bone distribution in successive 20 cm deep intervals at North Quarry.**

The variations in bonebed thickness appear to reflect undulations in the underlying sandstone. These differences are reflected in slight variations in large bone distribution. An example of this is the higher concentration of large bones in the western part of North Quarry (Fig 5).

**Element Orientation**

Element orientation of 268 long bones from the four main quarries shows no significant directionality (p = 0.170) based on a Rayleigh test of uniformity (Fig 8a). Separating the bones by quarry also shows no directionality in the three largest quarries (North, p = 0.373, Fig 8b; South, p = 0.224, Fig 8c; Southeast, p = 0.142, Fig 8d), but the fourth quarry has a definitive mean directionality (Teague, p = 0.015, Fig 8e). However, due to the very close proximity of these four quarries, some other factor such as unevenness of the floor or small sampling size may have influenced the directionality of long bones as they were deposited. Further excavation in the quarries will hopefully clarify these possibilities. No plunge data were analyzed since most elements were found in horizontal or near-horizontal orientation.

**Fig 8. Long bone orientation in the main quarries. Outer lines are bootstrapped 95% confidence intervals.**

**Taxon Representation**

The HR Bonebed has produced a wide range of taxonomically diverse organisms. In terms of body size, Dinosauria is by far the largest taxon represented (>95%), with *E. annectens* providing the bulk of these elements, averaging about 94% in each of the four main quarries (Table 2). These data include all elements found that are identifiable taxonomically; this filter (identifiability) eliminates about 400 elements from the total recovered. The taxa represented are consistent among the different quarries.

The identifiable elements include nine genera of Saurischia and five of Ornithischia. Other non-dinosaurian terrestrial taxa represented include squamates and mammals. Additionally, there are a variety of aquatic creatures, represented by crocodiles, turtles, gar and other ray-finned fish, elasmobranchs, and both gastropod and bivalve mollusks. Thus, the HR Bonebed is multitaxic and monodominant [39].

Fish are known from scales, teeth, and vertebrae, each in about equal proportions, in the HR Bonebed. Gar are the most common fish (as in the Hell Creek sites [9]), but we have recovered only isolated teeth, vertebrae and scales of gars from the main quarries. Turtles were identified from broken carapaces and plastrons, as well as from isolated limb bones. *Leidyosuchus*, *Brachychampsa*, and perhaps other crocodylomorphs are recognized by the presence of teeth, scutes, some skull fragments and limb bones.

While the quarry is clearly dominated by the remains of Hadrosauridae, we do find some remains referable to Ceratopsidae, and a few skeletal elements assignable to Pachycephalosauridae, Nodosauridae, and Thescelosauridae dinosaurs (Table 2). Large and small theropods must have been present where the dead animals accumulated.  The shed teeth of theropods are common in the bonebed, as would be expected if they were scavenging the decaying *Edmontosaurus* carcasses. The most common of these pertain to the theropod families Tyrannosauridae, Dromaeosauridae, and Troodontidae.

In 2001, the remains of the foot of a *Nanotyrannus lancensis* were found on the surface in a site designated Stair Quarry (not included in this study). Over the next 15 years, excavation of the site yielded 50 additional bones from this specimen, including a right maxilla with teeth and a left dentary with teeth. Although not yet formally described, these remains have enabled us to clearly distinguish slender, blade-like shed teeth of *Nanotyrannus* from the more robust crushing teeth of *Tyrannosaurus,* both of which are commonly found in the bonebed.

Mammal fossils, identified only from teeth, are exceedingly rare in the bonebed. Several small, thin-walled hollow bones were also recovered that lacked proximal and distal ends.  These bones are probably from birds, a number of which have been recognized in the Lance/Hell Creek from more complete specimens [40].

**Age Distribution / Ontogenic Representation**

Lengths of complete long bones from the HR Bonebed are displayed in Fig 9. The almost continuous size range from approximately 50% of adult length to maximum adult length fits quite well into the adult and subadult categories of Ryan et al. [25]. In most categories, there is a single “outlier” which is at least 5% longer than the next longest bone. We removed this outlier and averaged the next three longest bones to get a defined maximum length for mature adult long bones. Following the definitions of Ryan et al. [25], looking closely at two-thirds and half this average element length should help differentiate adult, sub-adult and juvenile elements. However, there is no clear gap at the two-thirds length mark. There are no femora smaller than 50% the size of the largest femur in the bonebed. Tibiae, fibulae, humeri, ulnae, and radii include only one to a few elements slightly below 50% the size of the largest of each of these skeletal elements.

**Fig 9. Lengths of major long bones. Dark black bars denote the size of a bone 50% the length of the three longest specimens for each bone type (after the longest “outlier” is removed for each bone type [all categories except tibia]).**

**Element Distribution**

Through the 2016 field season, we have found 8,460 identifiable E. *annectens* skeletal elements in the HR Bonebed. The highest number of recovered bones were 114 pubes. Of these, 61 are left and nine are too incomplete to be certain which side they derive from. Thus, conservatively, 61 is the minimum number of individuals (MNI) for the assemblage (Table 3). Since there are approximately 389 elements in a hadrosaurid skeleton (lambeosaurine [1]), we would expect a complete bone count of 23,729. The 8,460 identifiable elements so far recovered from the HR quarries represent a 36% recovery rate. All percent totals above 36% are thus over-represented and those below 36% are under-represented. This overall high recovery rate is one of the benefits of a concerted long-term focus on a single bonebed. It allows us a more comprehensive view of the possible paleoecology and taphonomy for these creatures

**Table 3. Percentages of bones recovered based on MNI of 61, sorted by abundance.**

| Region | Element | % Total |  |  |  |  |
| --- | --- | --- | --- | --- | --- | --- |
| Cranial |  |  |  | Axial |  |  |
|  | Nasal | 66 |  |  | Rib | 68 |
|  | Quadrate | 62 |  |  | Subtotal: ribs | 46 |
|  | Jugal | 52 |  |  | Rib, cervical | 10 |
|  | Surangular | 46 |  |  | Subtotal: vert | 23 |
|  | Maxilla | 45 |  |  | Vertebra, cervical | 18 |
|  | Quadratojugal | 39 |  |  | Vertebra, caudal | 17 |
|  | Splenial | 38 |  |  | Vertebra, dorsal | 7 |
|  | Hyoid | 36 |  |  | Chevron | 15 |
|  | Pterygoid | 36 |  | Pectoral Girdle and Forelimb |  |  |
|  | Squamosal | 26 |  |  | Scapula | 66 |
|  | Premaxilla | 24 |  |  | Radius | 50 |
|  | Exoccipital | 21 |  |  | Humerus | 48 |
|  | Angular | 19 |  |  | Ulna | 39 |
|  | Ectopterygoid | 18 |  |  | Clavicle | 27 |
|  | Palatine | 16 |  |  | Metacarpal | 21 |
|  | Basisphenoid | 15 |  |  | Phalanx, manus | 18 |
|  | Alisphenoid | 12 |  |  | Ungual, manus | 14 |
|  | Parietal | 9 |  |  | Coracoid | 8 |
|  | Articulated Braincases | 7 |  |  | Carpal | 2 |
|  | Predentary | 2 |  | Pelvic Girdle and Hindlimb |  |  |
|  | Articular | 1 |  |  | Pubis | 100 |
| Orbital |  |  |  |  | Ischium | 85 |
|  | Postfrontal | 30 |  |  | Fibula | 67 |
|  | Lacrimal | 23 |  |  | Femur | 49 |
|  | Prefrontal | 21 |  |  | Tibia | 46 |
|  | Frontal | 18 |  |  | Ilium | 27 |
|  | Postorbital | 14 |  |  | Metatarsal | 25 |
| Mandible |  |  |  |  | Phalanx, pes | 16 |
|  | Dentary | 96 |  |  | Astragalus | 15 |
|  |  |  |  |  | Calcaneum | 11 |
|  |  |  |  |  | Ungual, pes | 11 |
|  |  |  |  |  | Sacrum | 9 |

Percentages of both recovered postcranial and cranial elements are illustrated for a single skeleton in Figures 10 and 11, respectively. We found no major differences between the relative representation of cranial versus postcranial elements, suggesting that decomposition took place at the same time and place. For the postcranial appendages, numbers of recovered elements decrease in a proximal to distal progression. Pubes and ischia in the pelvic girdle and scapulae in the pectoral girdle are the most commonly recovered appendicular elements. Major long bones are next in number, followed by more distal elements.

**Fig 10. Relative number of *Edmontosaurus* postcranial bones found that are over- (larger) or under- (smaller) represented compared to a 36% overall recovery rate in the bonebed.**

**Fig 11. Relative number of *Edmontosaurus* cranial bones found that are over- (larger) or under- (smaller) represented compared to a 36% overall recovery rate in the bonebed.**

Considering the skull (Fig 11) as an entity separate from the body, we would assume disarticulation to reach a similar extent as that of the rest of the body during decomposition. In the body, both the core and distal elements are found least frequently, whereas “intermediate elements” are found most frequently; these are also the largest skeletal elements in the body. This pattern is replicated in the skull. The small, “core” elements (of the braincase and immediately surrounding the braincase) and small elements located farthest away from the “core” of the skull (premaxilla and predentary) are found least frequently, whereas larger cranial elements (i.e., the dentary, nasal, and quadrate) are found most commonly, with intermediate-sized bones falling in between in terms of abundance.

**Weathering**

Bones in each of the HR quarries are uniform in both type of bone modification and comparative percentiles within each category of modification (Table 4). Data presented here are combined data from the four largest quarries, North (N), South (S), Southeast (SE), and Teague (T). Teeth, tendons and unidentified bone fragments have been left out of these evaluations.

**Table 4. Bone surface modifications and fracturing at the four largest HR quarries. Weathering, abrasion, and fracturing categories are adapted from Ryan et al., 2001.**

| Count | | | | | | | |
| --- | --- | --- | --- | --- | --- | --- | --- |
| Feature | Stage/style | N | S | SE | T | Total | Percent |
| Weathering | 0 | 2857 | 1921 | 1307 | 379 | 6464 | 98.9 |
|  | 1 | 17 | 15 | 18 | 2 | 52 | 0.8 |
|  | 2 | 7 | 0 | 1 | 1 | 9 | 0.1 |
|  | 3 | 3 | 0 | 4 | 1 | 8 | 0.1 |
| Abrasion | 0 | 2966 | 1988 | 1371 | 406 | 6731 | 97.6 |
|  | 1 | 43 | 34 | 20 | 12 | 109 | 1.6 |
|  | 2 | 27 | 10 | 8 | 2 | 47 | 0.7 |
|  | 3 | 4 | 3 | 2 | 0 | 9 | 0.1 |
| Fracturing | Longitudinal | 75 | 55 | 19 | 9 | 158 | 3.2 |
|  | Transverse | 553 | 101 | 72 | 5 | 731 | 14.7 |
|  | Greenstick/spiral | 727 | 634 | 494 | 204 | 2059 | 41.3 |
|  | Indeterminate | 66 | 61 | 75 | 14 | 216 | 4.3 |
|  | Unfractured | 716 | 674 | 353 | 65 | 1808 | 36.4 |

**Abrasion**

The four stages defined by Ryan et al. [25] were applied to the 6,896 elements analyzed for abrasion. 97.6% of these elements exhibit stage 0, showing little or no abrasion (Table 4). Those exhibiting stage 1 (with broken edges that are slightly rounded) represent about 1.6% of the total. Rarely do any of the bones exhibit stage 2 (0.7%) or stage 3 (0.1%) abrasion. Although they are not included in these calculations, the majority of bone fragments also show little to no abrasion. They usually exhibit well-defined edges with very little or no rounding observable.

**Breakage and Fracturing**

We have added the “indeterminate” category to the three major categories of fracturing (longitudinal, transverse, and spiral/greenstick) used by Ryan et al. [25]. We then applied these definitions to the 4,972 bones analyzed for breakage and fracturing. The most abundant category, greenstick/spiral (41%), is commonly seen in fresh bone. However, about 35% of bones collected from the HR Bonebed are unfractured (Table 4). This is in contrast to many other hadrosaur bonebeds around the world which report single digit percentages of unbroken bones (Eastern Russia [41]; Utah, USA [42]: Alberta, Canada [43]).

**Discussion**

**Element Distribution and Transport**

The significance of element distribution in disarticulated bonebeds has been debated ever since Voohries [44] created three categories of transport for mammalian elements (primarily sheep and coyote) in 1969. His Group I bones were transported by saltation or flotation and included ribs, vertebrae, sacra, and sterna. Those in Group II were transported by traction and included femora, tibiae, humeri, pelves, radii, and metapodia. Those elements that moved very little, the skull and mandibles, he placed in Group III. Light elements were transported over larger distances than heavier ones [44]. Interestingly, scapulae, phalanges, and ulnae were lumped as intermediary between groups I and II. Thus, he fully recognized the challenges of defining few, specific categories.

Behrensmeyer [45] broadened the data available by using bones from a wider variety of animal species including hippopotamus, zebra, antelope, crocodile, and fish. She found that density appeared to be more important than size in determining whether or not bones will disperse. Shape was an important factor only for particular bones. The Voorhies groups were updated by Lehman [46], and used by Ryan et al. [25] for *Centrosaurus* and Bell and Campione [43] for *Edmontosaurus*, by adding ischia and metapodia to group I, reassigning ribs and dorsal vertebrae to group II, and reassigning sacra and scapulae (from group I), as well as femora, tibiae, and humeri (from group II), into group III.

Because Voorhies groups are based on extant animal elements and density plays such an important role in transport, and because we can never know the native densities for bones of extinct dinosaurs, the blind application of Voorhies categories is misguided. Gangloff and Fiorillo [6] did not find these categories useful for the Liscomb bonebed but attributed that to the highly fragmented nature of skeletal elements in that assemblage. Fiorillo et al. [47] found that the majority of elements they recovered were from Voorhies group II, and that heavy elements were over-represented while light elements were under-represented. This appears to be a common finding in many quarries containing disarticulated skeletons of centrosaurines [25] and hadrosaurids [41, 43, 48].

Britt et al. [42] suggested using shape (block, rod, flat, complex) as a different solution to this problem of transportation and winnowing. However, they found that this categorization was an imperfect solution since some shapes were still under-represented (block), and others were over-represented (complex).

The large number of elements found in the HR Bonebed helps clarify this perplexing problem. A very interesting pattern is emerging, in that core elements of the torso, vertebrae in general, and sacra in particular, are under-represented. Ilia are also much lower in number than expected based on the other pubic and pectoral girdle elements. This is perhaps because the ilia are more tightly bound to the sacrum than they are to either the pubis or ischium and may be transported together at a different rate than elements of different density or size. Pubic and pectoral girdle bones are the most commonly found bones in the HR Bonebed, followed closely by major long bones. The number of recovered bones decreases the more distal they are in the skeleton. It appears that, in general, axial and distal elements are under-represented, and long bones are over-represented. Ribs are well-represented, but one rib may be counted more than once if two fragments of the same rib are counted as two elements.

In 1970, Sternberg [11] reported a similar observation concerning element numbers and suggested that the dorsal and proximal caudal vertebrae were held together more tightly due to ossified tendons predominating in these regions of the spinal column. This can be seen in a juvenile *Edmontosaurus* [49], and may represent a separate class of transport than long bones. But, since most reported *Edmontosaurus* bonebeds are composed of disarticulated elements, it is difficult to generalize. Limited preservation of axial elements is a widespread phenomenon problem even in the earliest known dinosaurs [50]. Head, neck, hand and foot bones, and distal caudal vertebrae appear to have disarticulated most readily.

In the HR Bonebed, the dentary is the second-most commonly recovered element. It is relatively large and heavy like the major limb bones, and probably disarticulated readily. Major body parts (forelimb, lower jaw, cranium, hindlimb) with synovial joints disarticulate rapidly (weeks to months) during decomposition [51]. If disarticulation followed decomposition, the “outer” bones would dissociate first. Since hadrosaurid skulls do not exhibit fusion outside the braincase [52], the braincase and closely-associated elements would stay together and act as a single element during transport. This may mirror the tightly bound sacrum and dorsal vertebrae, resulting in a slightly different rate of transport than the long bones of the body.

Loss of small elements can occur through a variety of processes. Small scavengers can remove peripheral, quickly disarticulating elements, but this is unlikely for the HR Bonebed. Less than 4% of our bones exhibit identifiable tooth marks even though we have recovered a number of theropod teeth. Likewise, attrition attributable to weathering seems unlikely due to the low percentage of weathered elements and the similarity in preservation quality between large and small elements. If the carcasses experienced a bloat and float scenario, distal elements would be removed first, as seen in turtles [53], crocodiles [54], Ichthyopterygia [55], and *Serpianosaurus* [56]. This could account for the low recovery rate of distal appendage elements, but not for the low axial element recovery rate.

**Bonebeds and Taphonomy**

Bonebeds comparable to the HR Bonebed contain primarily disarticulated elements [42] or completely or almost completely disarticulated elements [3, 6, 41, 43, 57]. A similar biotic or abiotic mechanism may be at work in all these examples [58]. Biotic factors such as carnivory appear to be rare in the literature, but teeth marks have been reported to be common in some *Edmontosaurus* assemblages [43]. Alternatively, the relative decomposition and disarticulation of carcasses can be used to estimate the duration of surface exposure [24]. Extremely limited articulation in the bonebed suggests that a mass mortality event occurred followed by almost complete disarticulation prior to transport and burial.

A multimodal orientation of long bones also appears to be common in other dinosaur bonebeds. For example, a weak bimodal distribution NW to SE and SW to NE is seen in bonebeds BB030 and BB091 from the Dinosaur National Park region in Canada [1, 59]. Similar orientations are also seen in the Liscomb Bonebed in Alaska [6], the Dalton Wells Bonebed in Utah [42, 60], and a tendency to line up in this orientation in the Blagoveschensk Bonebed in Russia [41]. Both Phillips [61] and Gangloff and Fiorillo [6] conclude that long bones were oriented either parallel or perpendicular to the prevailing current. Weeks [13] found a general southeasterly orientation on crossbed dip directions in the region surrounding the HR quarries. This mirrors the paleocurrent direction for the Lance Formation in the Powder River Basin [14] and is reflected in the bones from Teague Quarry when taken alone. However, the small number of elements recovered in this quarry may introduce a sampling bias since there is no significant orientation to bones within the HR Bonebed as a whole. As more area is excavated, variations in element orientation may lead us to better understand the dynamics of this debris flow.

The rich diversity of taxa found in the HR Bonebed mirrors that found in the Dinosaur Park Formation [25] but it does not have as high a percentage of theropod elements and differs with respect to the primary monodominant species. At the Hanson Ranch, the ratio of theropod elements to those of *Edmontosaurus* is slightly less than 4%, giving a predator to prey ratio of about 1:20. The ratios in other *Edmontosaurus* bonebeds range from about 1:50 in the Liscomb Bonebed [6] and 1:10 based on NISP in the Dalton Wells Bonebed [42] to an approximately 1:6 ratio based on NISP in the Danek Bonebed [43]. Considering that trophic level energetics are about 1:10, it appears that predators are under-represented in the HR Bonebed. However, Bakker [62] suggested that 3% for dinosaur predators is a likely realistic figure.

Determining an age profile of dinosaurs is difficult as little is known about actual growth rates. If taphonomic processes, specifically post-depositional transport during a catastrophic event, have not altered the size-frequency distribution, bone length categories should represent animal sizes of the original population [63]. Histological criteria help clarify growth rate, but the lines of arrested growth (LAGs) are still inconclusive in determining exact yearly intervals of bone growth [64 – 66].

Bonebeds which are spread over a broad geographical area may likely represent catastrophic events enveloping a standing population, enabling characterization of age segregation if a species employs herding as a life strategy. A wide range of lengths have been reported for long bones in hadrosaur bonebeds. Brinkman et al. [63] found three distinct age clusters compiled from many quarries in the Dinosaur Park Formation. Long bones found at the Standing Rock Hadrosaur Site, South Dakota [9] and the Danek Bonebed in Alberta [43] also represented different age classifications but there were no very small sizes and the most common categories were “subadult” and “adult” based on Horner et al. [64]. Recently, Wosik [67] combined size-frequency distributions and long bone histology in elements of *Edmontosaurus* from the Dinosaur Park Formation. He found four peaks in size that correlated with osteohistological analyses, suggesting that full adult body size was attained in 5 years.

Other bonebeds are limited to a much narrower range of length for long bones. Varricchio and Horner [69] found that several monodominant bonebeds from the Upper Cretaceous Two Medicine Formation consist of a single subadult size class of hadrosaurs 3 – 3.5 m long. They interpreted these animals as belonging to a first-year age class, suggesting that juvenile growth rates were as rapid as large ungulates today. The Sun River Bonebed inters primarily “late juveniles” with no adult material [48]. Similarly, long bones from the Prince Creek formation in Alaska were best described as belonging to “late juveniles”, suggesting the possibility of age-segregation [6]. This inference has been reinforced by the discovery of bonebeds with long bones limited to 50 – 100% of maximum adult length, falling into the categories of “sub-adult” and “adult” [41]. These same limits are mirrored in the HR Bonebed.

Ryan et al. [25] presented a simplified three-level approach to age-classes defining *Centrosaurus apertus* based on percentage of adult long-bone length: Juvenile – less than half of adult size, Sub-adult – half to two-thirds an adult, and Adult (typical large, “adult” museum specimen). Histological data shows that LAGs are rare in bones of individuals less than 3.5 m in length (approximately 50% adult length) [64]. Complicating these criteria are the challenges of sexual dimorphism and the taxonomic descriptions of species based on possible ontogenetic differences. Variations in adult size may therefore potentially reflect individual or gender variation and not relative age [63, 68].

Taphonomic variability can also impact bonebed diversity. If a thanatocoenosis formed just prior to the reproductive season, it would likely not contain the bones from young animals [41]. In the Blagoveschensk Dinosaur Locality (BDL) bonebed, very small elements from older individuals were preserved, leading the authors to conclude that the absence of elements from young individuals is representative of the living population at that time [41]. Keeping in mind individual and gender size differences in a typical group of animals, and the rapid first-year growth postulated by Varicchio and Horner [69], we likewise hypothesize that the HR bonebed is representative of an *Edmontosaurous* herd just prior to seasonal breeding. We have found small adult bones of many different species, but not from *Edmontosaurus.* If histological and other aging techniques were to demonstrate that the smallest individuals are older than 1 year, it is likely that younger individuals remained separated at some other place, possibly in juvenile groups.

Bone modifications help us understand the taphonomic changes that occurred after death. Of the 6,533 elements examined for weathering, the vast majority (98.9%) fall within stage 0 (Table 3). Of the remaining bones, 0.8% show stage 1 weathering and both stages 2 and 3 have only 0.1% recorded. Behrensmeyer [24] suggested that exposed bones develop Stage 1 cracking within a few months. Limited skeletal element abrasion suggests that these elements were not exposed to protracted transport because either: a) they were not moved very far, or; b) that they were light and did not forcefully impact the surrounding debris flow elements or surfaces over which they travelled. Behrensmeyer [70] showed that in natural rivers, unweathered bones can travel many kilometers without significant rounding. For the HR Bonebed, a more plausible possibility is that these elements were transported in a viscous matrix, so they would not be subject to the bumping or abrasion that would round edges. The total distance travelled might be considerably farther than if in a more fluid matrix.

Of those bones exhibiting fracturing and breakage, the large percentage of spiral/oblique fractures elicits three possible explanations, all of which would need to occur fairly rapidly postmortem. There may have been significant scavenging, but only about 2% of recovered skeletal elements exhibit distinct tooth marks, limiting this possibility as an explanation for the common fracturing of skeletal elements. Secondly, one might expect some breakage could occur during hydraulic transport. However, a debris flow over a silty/sandy substrate would most likely not result in heavy abrasion [59], which could lead to breakage. Behrensmeyer [24, 70] reported that no breakage occurred when fresh large mammal bones were transported over several kilometers in natural rivers. Very little abrasion is recorded for HR Bonebed specimens (Table 3), making transport an unlikely source of breakage for these bones. The third possibility, trampling, seems to be the most likely cause of spiral/greenstick breakage in these bones. Assuming the fine sediment they are buried in was the same on which they died, this type of breakage could easily have happened with minimal abrasion occurring [71 – 72].

Eberth and Getty [59] suggest that there is no necessary link between scavenging and trampling. Since such a large percentage of intact and spirally fractured elements occur in the HR Bonebed, with limited scavenging, it is possible that fracturing was caused by large dinosaurs walking across areas of freshly concentrated bones. This correlates well with the taphonomy of hadrosaurs found in the Princess Bonebed at Dinosaur National Park [1].

The pristine condition of these bones and the uniformity across all quarries suggests that these specimens originated from a single catastrophic event, as opposed to an attritional assemblage over time [39, 71]. They were probably not exposed for more than a short period of time and were exposed to minimal post-depositional weathering.

**Depositional Model**

Depositional environments that have been ascribed to the Lance include meandering and braided streams [73] in western Wyoming, and marginal marine and fluvio-deltaic [74], floodplain [75], or lacustrine [17] settings in central Wyoming. To the south, the Lance is attributed to fluvio-deltaic processes [18]. In the northeast, the beds are described as being of marine origin [76], but as a whole, the Lance is generally regarded as being of fluvial origin [77]. However, the Lance Formation exhibits a diversity of sedimentary characteristics across the extent of its outcrops [13, 77]. Much of the exposed stratigraphy in the study area on the eastern limb of the Powder River syncline consists of from one- to five-meter-thick beds of white, immature, fine grained quartz sandstone, alternating with meter scale gray to black, poorly indurated shaly siltstone to claystone. The sandstone bedding, where preserved, displays large and medium scale tabular and trough crossbedding. The sandstones often show severe disruption of bedding planes, apparently caused by soft sediment deformation during potential seismic disturbances (Fig 2; [78]), Where this disturbance is evident, underlying beds of sandstone may be unaffected. This suggests the affected sand units were subjected to a massive seismic event before dewatering could occur. This is more readily accommodated in a marine or marginal marine setting where dewatering may be delayed for up to six months [79]. Because these features are present at numerous horizons within the Lance, the seismic disturbance could not be directly attributable to a single event such as, for instance, an asteroid impact, and the explanation must be sought in other tectonic processes such as mountain building, perhaps associated with the Laramide orogeny [77]. The transport direction based on paleocurrents in the sandstones indicates the source area (and possibly the paleoslope) was from the west and northwest [14, 80].

The bones in the HR Bonebed occur everywhere as a normally graded sequence within the shaly mudstone. The sediment surrounding the bones is often a claystone with a particle size many orders of magnitude smaller than the largest bones encased in it. Depositing such an assemblage in a fluvial environment is problematic [81] and the bonebed exhibits characteristics of a submarine debris flow [13, 82] perhaps as part of a shallow marine delta front [83]. Other authors have likewise attributed similar Cretaceous bonebeds to debris flows of various types [9, 42, 60, 71, 84 - 85]. While this bonebed shares some of the features identified by Britt et al. [42], it also has significant differences. It is a tabular deposit, probably broadly lenticular, and elements are generally concentrated near the bottom of the unit. This deposit differs from the criteria of Britt et al. [42] in being consistently free of pebbles or cobbles and being generally well graded with respect to bones. It also does not rest on a paleosol, and most elements are from adult animals and are not broken prior to burial. Aquatic vertebrates are also relatively common. The upper boundary of the bonebed unit is in sharp and planar contact with the overlying sandstone. Such a contact is also more readily explained in a marine environment than in a fluvial setting, where a change from claystone deposition to sandstone would be more likely to produce an erosive contact.

In our opinion, the best explanation for the transport and deposition of the bones and the containing sediment is that they were carried together as part of a seismically activated subaqueous debris flow originating from an oversteepened prograding delta an unknown distance to the west. The aerial extent and concentration of bones in the unit would seem to constrain the transport distance to the order of kilometers or less. Because the bones exhibit very little transport-inducedabrasion, the flow must have been matrix-supported.

The scenario surrounding the demise of these animals and the formation of this deposit appears to be very similar to that depicted for other Upper Cretaceous *Edmontosaurus* bonebeds [3, 9, 42 - 43, 48]. A large herd of *Edmontosaurus* died, either by drowning, or by some other mass catastrophic process. The carcasses may have bloated and floated for a period of time, but eventually their remains became stranded and accumulated in a nearshore environment. Here, they were subjected to scavenging by various carnivores, including tyrannosaurids, dromaeosaurids, and troodontids while decay occurred. If the habits of modern predators/scavengers were operative in the Cretaceous, the food target of choice would be the ribcage and the soft organs inside [87]. Since dinosaurs had a more extensive ribcage than modern mammals, accessing the organs may have entailed biting through the ribs. This could explain why the ribs, which are generally resistant to transport breakage, are so often found in pieces with edges that exhibit shearing and scoring.

Aerial exposure was limited to a period of time long enough (weeks to months) to result in decay-mediated disarticulation of the skeletons but was not long enough (months to years) for weathering to affect the bones. At this point, it appears probable that a significant fluvial flood or a regional seismic event initiated the resuspension of the underlying sediments enveloping the mass of disarticulating skeletons and transporting the mixture basinward within a debris flow. This resulted in the formation of the normally graded bonebed in a matrix of clay and silt.

**Comparison of Bonebeds**

Over the past few decades, authors describing hadrosaur bonebeds have started including data on element representation [1, 3 - 4, 6, 9, 48]. There is currently no formal protocol, as some authors have grouped elements into various categories such as hindlimb long bones, forelimb bones [1], specific elements as defined in Voorhies groups [3], or more complete lists of individual bones [6, 9, 48]. Those with comparable data are included in Table 4. Correlations with HR Bonebed elements are presented in Fig 12 and Table 5.

**Fig 12. Correlations of percent MNI among published hadrosaurid bonebeds. The closer the individual dots are to the line, the more significant the correlation is. The gray dotted lines indicate the 95% confidence intervals around each black best-fit line.**

**Table 5. Element comparisons among different bonebeds. All except East Fork River are dinosaurs. Percent of total possible elements based on each bonebed MNI are listed. Percent of recovered extant bones are listed for East Fork River. Data for the other sites were derived from the following references: Standing Rock [9], Sun River [48], Blagovenschensk[41], Liscomb[6], Horseshoe Canyon [3], Mason Dinosaur Quarry [10], East Fork River [90]. Abbreviations: MNI, minimum number of individuals; Vert., vertebra.**

| Region | Element | Hanson Ranch | Standing Rock | | Liscomb | | Sun River | Horseshoe Canyon | Blagovenschensk | Mason | East Fork River |
| --- | --- | --- | --- | --- | --- | --- | --- | --- | --- | --- | --- |
|  | #Elements | 8460 | 4702 | 2769 | | 537 | | 1612 | 1500 | 3000 | 311 |
|  | Basisphenoid | 15 | 16 | 37 | |  | |  |  |  |  |
|  | Exoccipital | 21 | 13 | 21 | | 19 | |  |  |  |  |
|  | Jugal | 52 | 57 | 37 | | 6 | |  | 19 |  |  |
|  | Maxilla | 45 | 51 | 11 | | 31 | |  |  | 98 |  |
|  | Nasal | 66 |  |  | | 6 | |  |  |  |  |
|  | Parietal | 9 | 11 | 16 | |  | |  |  |  |  |
|  | Premaxilla | 24 |  |  | | 25 | |  |  |  |  |
|  | Quadrate | 62 | 61 | 66 | | 38 | |  |  |  |  |
|  | Squamosal | 26 |  |  | | 6 | |  |  |  |  |
|  | Frontal | 18 | 43 | 13 | | 25 | |  |  |  |  |
|  | Postfrontal | 30 |  |  | | 13 | |  |  |  |  |
|  | Postorbital | 14 | 31 | 26 | | 13 | |  |  |  |  |
| Mandible | Dentary | 96 | 43 | 76 | | 75 | | 41 | 78 | 85 | 69 |
| Axial | Vert., caud | 9 | 41 | 31 | | 3 | | 5 |  |  |  |
|  | Vert., cerv | 18 |  |  | | 2 | | 1 |  |  |  |
|  | Vert., dors | 7 | 7 | 3 | | 9 | | 51 |  |  | 40 |
|  | Chevron | 15 |  |  | | 4 | |  |  | 20 |  |
|  | Rib | 68 |  |  | | 50 | | 25 |  | 47 | 6 |
|  | Rib, cervical | 10 |  |  | | 3 | |  |  |  |  |
|  | Clavicle | 27 |  |  | | 31 | |  |  |  |  |
|  | Coracoid | 7 |  |  | |  | | 23 |  | 29 |  |
|  | Humerus | 48 |  |  | | 69 | | 9 | 93 | 41 | 78 |
|  | Metacarpal | 21 | 71 | 29 | | 13 | | 3 |  | 45 |  |
|  | Phalanx, manus | 11 | 14 | 6 | | 2 | |  |  | 24 | 21 |
|  | Radius | 50 | 48 | 61 | | 56 | | 0 | 17 | 34 | 62 |
|  | Scapula | 65 | 32 | 34 | | 50 | | 0 | 26 | 54 | 33 |
|  | Ulna | 39 |  |  | | 44 | | 0 | 32 | 39 |  |
|  | Ungual, manus | 7 |  |  | | 2 | |  |  |  |  |
| Pelvic | Astragalus | 15 |  |  | | 6 | |  |  | 17 | 50 |
|  | Femur | 49 |  |  | | 63 | | 32 | 65 | 59 | 50 |
|  | Fibula | 67 |  |  | | 69 | | 36 | 56 | 83 |  |
|  | Ilium | 27 |  |  | | 50 | | 86 | 11 | 44 |  |
|  | Ischium | 85 |  |  | | 56 | | 91 | 19 | 100 |  |
|  | Metatarsal | 25 | 81 | 47 | | 25 | | 3 | 31 | 29 | 63 |
|  | Phalanx, pes | 13 | 15 | 7 | | 4 | |  |  | 24 | 0 |
|  | Pubis | 99 |  |  | | 63 | | 82 | 21 | 78 |  |
|  | Sacrum | 9 |  |  | |  | | 0 |  |  | 14 |
|  | Tibia | 46 | 38 | 71 | | 31 | | 23 | 56 | 76 | 50 |
|  | Ungual, pes | 4 |  |  | | 8 | |  |  |  |  |
| MNI |  | 61 | 44 | 36 | | 8 | | 11 | 36 | 20 | Varies |

There is a remarkable consistency in percentage of specific elements found across most of these bonebeds. Correlation between our percentages and those of Christians [ref#] (p = < 0.001), Gangloff and Fiorillo [ref #] (p = <0.001), Scherzer and Varricchio [ref#] (p = < 0.001), and Ullmann et al. [ref#] (p = 0.006), are all significant. There is even a significant correlation with extant animals as reported by Aslan and Behrensmeyer [ref#] (p = 0.012). The large number of bone fragments reported by Evans et al. [ref#] may explain why that bonebed in the Horseshoe Canyon Formation is not significantly correlated with the HR Bonebed (p = 0.142). These bonebeds are spread over a wide geographical area – Alaska, Alberta, Montana, South Dakota, and Wyoming. They also include ontogenically different ages with both adult/subadult ([9], this paper) and late juvenile animals [6, 48] as defined by Horner et al. [64]. The same taphonomic factors may be acting in all these bonebeds, since they share high percentages of large proximal limb bones (and the dentary) and low representation of small bones, the central vertebral column, and elements of the chondrocranium. Even in a distant bonebed [84], dentaries and humeri are the most abundant skeletal elements recovered. As more data become available, we hope the robustness of these comparisons can increase. It will also be interesting to see if this trend extends to other genera [88].

In our opinion, the most probable cause of HR Bonebed element distribution is that of hydraulic transport, deposition, and winnowing. Assuming most elements were disarticulated by decomposition prior to burial, a subsequent hydraulic event carried the bones some distance. Those elements that were denser (e.g., braincase, sacrum) were not moved as far as those that were smaller or more buoyant. Still-articulated elements (dorsal and caudal vertebrae) may have also resisted transport and acted as a single larger unit. This seems reasonable since we find the largest bones at the bottom of the bonebed and the smallest bones at the top. The erosion around our sites indicates that we are currently digging in what is likely the central portion of the HR Bonebed. We propose that few of the denser elements reached our portion of the bonebed during transport, and the smaller, less dense elements may have been carried afield beyond our current dig sites.

However, we cannot rule out the opposite possibility shown for mammalian elements, that some articulated skeletal elements are transported farther than when they are isolated [89 – 90]. Additionally, elements that have flesh (specific gravity ~1) still attached would float more easily than bone alone (specific gravity ~1.75). Larger articulated elements would be more likely to retain tissues and float, giving them a much different hydraulic character than isolated elements.

**Conclusions**

The Hanson Ranch Bonebed, exposed in five main and three exploratory quarries, is a monodominant hadrosaurid bonebed, consisting of sub-adult to adult *Edmontosaurus annectens*. Core body elements (vertebrae, sacra, braincases) and distal appendage elements (phalanges, carpals, predentaries) are recovered in low numbers. Proximal appendicular elements and dentaries are the most abundant bones in the assemblage. This suggests element transport and distribution based on size or relative weight. Comparisons with similar bonebeds across North America show a significant correlation in skeletal representation among sites, which implies they may share similar taphonomic histories. The most fitting scenario for the HR Bonebed is that of a large number of individuals dying in a single catastrophic event, followed by decomposition over weeks to months, because HR bones are generally in pristine condition. Shed theropod teeth suggest that scavenging occurred, but the large number of carcasses may be why there are very few tooth traces on bones. A subaqueous debris flow then moved the disarticulated elements far enough to permit vertical sorting of elements before coming to rest. The primarily silt and clay matrix must have been viscous enough and transport brief enough to provide protection from abrasion, but liquid enough for sorting and grading to have taken place. It is possible that this could have occurred in a marginal deltaic environment. According to Moore [28], a large majority (13 of 16) Upper Cretaceous dinosaur fossil assemblages are recognized as being deposited in a catastrophic setting, and this bonebed appears to follow that trend.

**Acknowledgments**

We thank the Hanson Ranch owners for their helpfulness in facilitating this research and Aaron Corbit for statistics and figure development.

**References**

1. Eberth DA, Evans DC, Lloyd DW. Occurrence and taphonomy of the first hadrosaurid bonebed from the Dinosaur Park Formation (Belly River Group, Campanian) at Dinosaur Provincial Park, Alberta, Canada, In: D.A. Eberth and D.C. Evans (Eds.), Hadrosaurs. Indiana University Press, Bloomington, Indiana. 2014:502-523.
2. Eberth DA, Bell PR. Stratigraphy of the Danek Bonebed (Upper Cretaceous Horseshoe Canyon Formation, central Alberta) and correlations with strata in the Drumheller and Grande Prairie regions. J Can Earth Sci. 2014;51(11):975-981. doi:0.1139/cjes-2014-0069.
3. Evans DC, Eberth DA, Ryan MJ. Hadrosaurid (*Edmontosaurus*) bonebeds from the Horseshoe Canyon Formation (Horsethief Member) at Drumheller, Alberta, Canada: geology, preliminary taphonomy, and significance. J Can Earth Sci. 2015;52:642-654.
4. Varricchio DJ. Taphonomy of Jack’s Birthday Site, a diverse dinosaur bonebed from the Upper Cretaceous Two Medicine Formation of Montana. Paleogeogr, Palaeoclimatol, Palaeoecol. 1995;114:297-323.
5. Colson MC, Colson RO, Nellermoe R. Stratigraphy and depositional environments of the upper Fox Hills and lower Hell Creek Formations at the Concordia Hadrosaur Site in northwestern South Dakota. Rocky Mountain Geology. 2004;9:93-111.
6. Gangloff RA, Fiorillo AR. Taphonomy and paleoecology of a bonebed from the Prince Creek Formation, north slope, Alaska. PALAIOS. 2010;25:299-317.
7. Tedesco LP, Halvorson HE, Cooper K. Mass mortality of a herd of hadrosaurs; Lance Creek Formation, east-central Wyoming, In: the Dinofest Symposium, D.L. Wolberg, K. Gittis, S. Miller, L. Carey and A. Raynor (Eds.). Acad Nat Sci, Philadelphia, Pennsylvania. 1998:60.
8. Chadwick A, Spencer L, Turner L. Preliminary depositional model for an Upper Cretaceous *Edmontosaurus* bonebed. J Vert Paleontol. 2006; Supplement 26:49A.
9. Ullmann PV, Shaw A, Nellermoe R, Lacovara KJ. Taphonomy of the Standing Rock Hadrosaur Site, Corson county, South Dakota. PALAIOS. 2017;32:779-796.
10. Christians JP. Taphonomy and sedimentology of the Mason Dinosaur Quarry, Hell Creek Formation (Upper Cretaceous), South Dakota. M.S. thesis, University of Wisconsin, Madison. 1992:91.
11. Sternberg CM. Comments on dinosaurian preservation in the Cretaceous of Alberta and Wyoming. Publications in Paleontology 4. National Museums of Canada, Ottawa. 1970.
12. Tedesco LP, Halvorsen HE, Cooper K. An *Edmontosaurus* Bone Bed: Lance Formation, East-central Wyoming. Unpublished manuscript. 1998.
13. Weeks SR. Depositional model of a late Cretaceous dinosaur fossil concentration, Lance Formation. M.S. thesis, Loma Linda University, California. 2016:123.
14. Conner CW. The Lance Formation: petrography and stratigraphy, Powder River basin and nearby basins, Wyoming and Montana. U.S. Geol Surv Bull. 1992;1917-I:17.
15. Seeland D. Laramide paleogeographic evolution of the eastern Powder River Basin, Wyoming and Montana. Wyoming Geological Association Guidebook, 39th Field Conference. 1988:29-34.
16. Ayers WB. Lacustrine and fluvial-deltaic depositional systems, Fort Union Formation (Paleocene), Powder River Basin, Wyoming and Montana. Am Assoc Pet Geol Bull. 1986;70(11):1651-1673.
17. Dickinson WR, Klute MA, Hayes MJ, Janecke SU, Lundin ER, McKittrick, et al. Paleogeographic and paleotectonic setting of Laramide sedimentary basins in the Rocky Mountain region: Geol Societ Am Bull. 1988:100(7):1023–1039.
18. Carvajal CR, Steel RJ. Thick turbidite successions from supply-dominated shelves during sea-level highstand. Geology. 2006;34:665-668.
19. Reynolds MW. Influence of recurrent Laramide structural growth on sedimentation and petroleum accumulation, Lost Soldier Area, Wyoming. Am Ass Pet Geol Bull. 1976;60(1):12-33.
20. Derstler K. The Dragon’s Grave – an *Edmontosaurus* bonebed containing theropod eggshells and juveniles. Lance Formation (Uppermost Cretaceous), Niobrara County, Wyoming. J Vert Paleontol, sup.1995;15:26A.
21. Leiggi P, Schaff CR, May P. Macrovertebrate collecting. In: Leiggi P, May P. (Eds.). Vertebrate Paleontological Techniques, Cambridge University Press, Cambridge. 1994:59-92.
22. Chadwick A, Silver M, Turner L, Woods J. The application of digital reconstruction techniques in taphonomy of an Upper Cretaceous dinosaur site in eastern Wyoming. J Taphonomy. 2016;4(1):1-14.
23. Jennings DJ, Hasiotis ST. Taphonomic analysis of a dinosaur feeding site using Geographic Information Systems (GIS), Morrison Formation, southern Bighorn Basin, Wyoming, USA. PALAIOS. 2006;27:480-492.
24. Behrensmeyer AK. Terrestrial vertebrate accumulations. In: Allison, P.A. Briggs, D.E.G. (Eds), Taphonomy: Releasing the data locked in the fossil record. Plenum, New York. 1991;291-335.
25. Ryan MJ, Russell AP, Eberth DA, Currie PJ. The Taphonomy of a *Centrosaurus* (Ornithischia: Ceratopsidae) bone bed from the Dinosaur Park Formation (Upper Campanian), Alberta, Canada, with comments on cranial ontogeny. PALAIOS. 2001;16:482-506.
26. Eberth DA, Rogers RR, Fiorillo AR. A practical approach to the study of bonebeds, In:Rogers RR, Eberth DA, Fiorillo AR (Eds.), Bonebeds: genesis, analysis, and paleobiological significance. University of Chicago Press, Chicago. 2007a:265-332.
27. Lyman RL. Quantitative Paleozoology. Cambridge University Press, Cambridge, UK. 2008;372.
28. Moore JR. Do terrestrial vertebrate fossil assemblages show consistent taphonomic patterns? PALAIOS. 2012;27:220-234.
29. Fiorillo AR. Aspects of bone modification applied to time resolution in the fossil record – an example from the Miocene of western Nebraska. Current Research in the Pleistocene. 1988;5:103-109.
30. Behrensmeyer AK. Taphonomic and ecologic information from bone weathering. Paleobiol. 1978;4:150-162.
31. Haynes G. Frequencies of spiral and green-bone fractures on ungulate limb bones in modern surface assemblages. Am Antiq. 1983;48:102-114.
32. Campione NE, Evans DC. Cranial growth and variation in edmontosaurs (Dinosauria: Hadrosauridae): implications for the latest Cretaceous megaherbivore diversity in North America. PLoS ONE. 2011;6:e25186.
33. Xing H, Mallon JC, Currie ML. Supplementary cranial description of the types of *Edmontosaurus regalis* (Ornithischia: Hadrosauridae), with comments on the phylogenetics and biogeography of Hadrosaurinae. PLoS ONE. 2017;12(4):e0175253.doi.org/10.1371/journal.pone.0175253
34. Paul GS. The Princeton field guide to dinosaurs. 2nd ed. Princeton University Press. Princeton, UK. 2016:360.
35. Berner RA. Calcium carbonate concretions formed by the decomposition of organic matter. Science. 1968;159:195-7. doi: 10.1126/science.159.3811.195
36. Yoshida H, Yamamoto M, Minami M, Katsuta N, Sin-ichi S, Metcalfe R. Generalized conditions of spherical carbonate concretion formation around decaying organic matter in early diagenesis. Sci Rep. 2018;8:6308. doi: 10.1038/s41598-018-24205-5
37. Chiba K, Ryan MJ, Braman DR, Eberth DA, Scott EE, Brown CM, et al.. Taphonomy of a monodominant *Centrosaurus apertus* (Dinosauria: Ceratopsia) bonebed from the Upper Oldman Formation of southeastern Alberta. PALAIOS. 2015;30:655-667.
38. Woodward HN, Freedman Fowler EA, Farlow JO, Horner JR. *Maiasaura*, a model organism for extinct vertebrate population biology: a large sample statistical assessment of growth dynamics and survivorship. Paleobiology. 2015;1(4):503-527.
39. Eberth DA, Shannon M, Nolan BG. A bonebeds database: Classification, biases, and patterns of occurrence, In: R.R. Rogers, D.A. Eberth, and A.R. Fiorillo (Eds.), Bonebeds: genesis, anlaysis, and paleobiological significance. University of Chicago Press, Chicago. 2007b:103-221.
40. Longrich NR, Tokaryk T, Field DJ. Mass extinction of birds at the Cretaceous–Paleogene (K–Pg) boundary. PNAS. 2011;108(37):15253-15257.
41. Lauters P, Bolotsky YL, Itterbeeck JV, Godefroit P. Taphonomy and age profile of a latest Cretaceous dinosaur bone bed in far eastern Russia. PALAIOS. 2008;23(3):153-162.
42. Britt BB, Eberth DA, Scheetz RD, Greenhalgh BW, Stadtman KL. Taphonomy of debris-flow hosted dinosaur bonebeds at Dalton Wells, Utah (Lower Cretaceous, Cedar Mountain Formation, USA). Palaeogeogr, Palaeoclimatol, Palaeoecol. 2009;280:1-22.
43. Bell PR, Campione NE. Taphonomy of the Danek Bonebed: a monodominant *Edmontosaurus* (Hadrosauridae) bonebed from the Horseshoe Canyon formation, Alberta. Can J Earth Sci. 2014;51:1-15.
44. Voohries MR. Taphonomy and population dynamics of an early Pliocene vertebrate fauna, Knox County, Nebraska. University of Wyoming Contributions to Geology Special Paper no. 1. 1969;1-69.
45. Behrensmeyer AK. The taphonomy and paleoecology of Plio-pleistocene vertebrate assemblages east of Lake Rudolf, Kenya. Bull Mus Comp Zool. 1975;146: 473-578.
46. Lehman TM. A ceratopsian bonebed from the Aguja Formation (Upper Cretaceous), Big Bend National Park, Texas. M.S. thesis, University Press, New York. 1982.
47. Fiorillo AR, Padian K, Musikasinthorn C. Taphonomy and depositional setting of the Placerias Quarry (Chinle Formation: Late Triassic, Arizona). PALAIOS. 2000;15:373-386.
48. Scherzer BA, Varricchio DJ. Taphonomy of a juvenile lambeosaurine bonebed from the Two Medicine Formation (Campanian) of Montana, United States. PALAIOS. 2010;25:780-795.
49. Wosik M, Goodwin MB, Evans DC. A nestling-sized skeleton of *Edmontosaurus* (Ornithischia, Hadrosauridae) from Hell Creek Formation of northeastern Montana, U.S.A., with an analysis of ontogenetic limb allometry. J Vert Paleontol. 2017;37:6,e1398168. doi 10.1080/02724634.2017.1398168
50. Muller RT, Pretto FA, Stefanello M, Silva-Neves E, Sias-Da-Silva S.. On a dinosaur axis from one of the oldest dinosaur-bearing sites world-wide. Acta Palaeontologica Polonica. 2017;62(3):543-548.
51. Lyman RL. Vertebrate taphonomy: Cambridge manuals in archaeology. Cambridge University Press, Cambridge. 1994.
52. Gates TA. The Late Jurassic Cleveland-Lloyd Dinosaur Quarry as a drought-induced assemblage. PALAIOS. 2005;20:363-375.
53. Brand LR, Hussey M, Taylor J. Taphonomy of freshwater turtles: decay and disarticulation in controlled experiments. J Taphonomy. 2003;1(4):233-245.
54. Syme CE, Salisbury SW. Patterns of aquatic decay and disarticulation in juvenile Indo-Pacific crocodiles (Crocodylusporosus), and implications for the taphonomic interpretation of fossil crocodyliform material. Palaeogeogr, Palaeoclimatol, Palaeoecol. 2014;412:108-123.
55. Beardmore SR, Furrer H. Evidence of a preservational gradient in the skeletal taphonomy of Ichthyopterygia (Reptilia) from Europe. Palaeogeogr, Palaeoclimatol, Palaeoecol. 2016;443:131-144.
56. Beardmore SR, Orr PJ, Manzocchi T, Furrer H, Johnson C. Death, decay and disarticulation: a method of modelling the skeletal taphonomy of marine reptiles demonstrated using *Serpianosaurus* (Reptilia; Sauropterygia). Palaeogeogr, Palaeoclimatol, Palaeoecol. 2012;337-338:1-13. doi: 10.1016/j.palaco.2012.03.018
57. Brouwers EM, Clemens WA, Spicer RA, Ager TA, Carter LD, Sliter WV. Dinosaurs on the North Slope, Alaska: High latitude, latest Cretaceous environments. Science. 1987;237:1608-1610.
58. Behrensmeyer AK. Bonebeds through time. In: Rogers RR, Eberth DA,Fiorillo AR (Eds.) Bonebeds: genesis, analysis, and paleobiological significance. The University of Chicago Press, Chicago. 2007;65-102.
59. Eberth DA, Getty MA. Ceratopsian bonebeds: occurrence, origins, and significance, In: Currie PJ, Koppelhus EB (Eds.), Dinosaur Provincial Park: A spectacular ancient ecosystem revealed. Indiana University Press, Bloomington, Indiana. 200;:501-536.
60. Eberth DA, Britt BB, Scheetz R, Stadtman KL, Brinkman DB. Dalton Wells: Geology and significance of debris-flow-hosted dinosaur bonebeds in the Cedar Mountain Formation (Lower Cretaceous) of Eastern Utah, USA. Paleogeogr, Palaeoclimatol, Palaeoecol. 2006;236:217-245.
61. Phillips RL. Depositional environments and processes in Upper Cretaceous nonmarine and marine sediments, Ocean Point dinosaur locality, North Slope, Alaska. Cretac Res. 2003;24:499-523.
62. Bakker RT. Dinosaur Bioenergetics – A Reply to Bennett and Dalzell, and Feduccia. Evol. 1974;28(3):497-503.
63. Brinkman DB, Eberth DA, Currie PJ. From bonebeds to paleobiology: applications of bonebed data, In: Rogers, R.R., Eberth, D.A. and Fiorillo, A.R. (Eds.) Bonebeds: genesis, analysis, and paleobiological significance. The University of Chicago Press, Chicago. 2007;221-263.
64. Horner JR, De Ricqles A, Padian K. Long bone histology of the hadrosaurid dinosaur *Maiasaura peeblesorum*: growth dynamics and physiology based on an ontogenetic series of skeletal elements. J Vert Paleontol. 2000;20:115-129.
65. Lee AH, Werning S. Sexual maturity in growing dinosaurs does not fit reptilian growth models. PNAS. 2008;105(2):582-587. doi: 10.1073/pnas.0708903105
66. Cooper LN, Lee AH, Taper ML, Horner JR. Relative growth rates of predator and prey dinosaurs reflect effects of predation. Proc Roy Societ. 2008;275:2609-2615.
67. Wosik M. Life history of hadrosaurids from the Dinosaur Park Formation of Alberta, Canada; Vertebrate Anatomy Morphology Palaeontology. Can Soc Vert Palaeontol. 2017:54. doi 10.18435/B5S3D
68. Griffin CT, Nesbitt SJ. Anomalously high variation in postnatal development is ancestral for dinosaurs but lost in birds. PNAS. 2016;113(5):14757-14762. doi/10.1073/pnas.1613813113
69. Varricchio D, Horner JR. Hadrosaurid and lambeosaurid bone beds from the Upper Cretaceous Two Medicine Formation of Montana: taphonomic and biologic implications. Can J Earth Sci. 1993;30:997-1006.
70. Behrensmeyer AK. Time resolution in fluvial vertebrate assemblages. Paleobiol. 1982;8:211-228.
71. Rogers RR. Taphonomy of three dinosaur bone beds in the Upper Cretaceous Two Medicine Formation of northwestern Montana: evidence for drought-related mortality: PALAIOS. 1990;5:394-413.
72. McLain M. Taphonomy of a Lance Formation (Maastrichtian, WY) Dinosaur Bonebed with a Focus on Tooth Traces. (Ph.D. Dissertation), Loma Linda University, Loma Linda, California, U.S.A. 2016.
73. Montgomery SL, Robinson JW. Jonah Field, Sublette county, Wyoming: Gas production from over-pressured upper Cretaceous Lance sandstones of the Green River basin. Am Assoc Pet Geol Bull. 1997;81:1049-1062.
74. Gillespie JM, Fox JE. Tectonically influenced sedimentation in the Lance Formation, eastern Wind River Basin, Wyoming. The Mountain Geologist.1991;28:53-66.
75. Breithaupt BH. Paleontology and paleoecology of the Lance Formation (Maastrichtian), east flank of Rock Springs Uplift, Sweetwater County, Wyoming. Rocky Mountain Geology. 1982;21(2):123-151.
76. Lloyd ER, Hares CJ. The Cannonball marine member of the Lance Formation of North and South Dakota and its bearing on the Lance-Laramie problem. J Geol. 1915;23(6):523-547.
77. Committee WGATS. Geologic history of Powder River Basin. Am Assoc Pet Geol Bull. 1965;49(11):1893-1907.
78. Weeks SR, Chadwick AV. A Regionally Extensive Lancian Seismite Serves as a Time Synchronous Stratigraphic Marker for Mapping Dinosaur Bonebeds in Northeastern Wyoming. Soc Vert Paleontol. 2012;32:191.
79. Talling PJ, Paull CK, Piper DJ. How are subaqueous sediment density flows triggered, what is their internal structure and how does it evolve? Direct observations from monitoring of active flows. Earth-Sci Rev. 2013;125:244-287.
80. Weeks LG. Origin of carbonate concretions in shales, Magdalena Valley, Colombia. Geol Soc Am Bull. 1967;68:96-102.
81. Reineck HE, Singh IB. Depositional Sedimentary Environments. Springer, Berlin, Heidelberg. 1980:551.
82. Prior DB, Bornhold BD, Johns MW. Depositional characteristics of a submarine debris flow. J Geol. 1984;92(6):707-727.
83. Bhattacharya JP. Deltas. Special Publication-SEPM. 2006;84:237-292.
84. Lauters P. Etude de population et taphonomie du gisement à hadrosauridés (Dinosauria: Ornithopoda) de Blagoveschensk, Russia. M.S. thesis, Université Libre de Bruxelles, Brussels. 2005:80.
85. Van Itterbeeck J, Bolotsky Y, Bultynck P, Godefroit P. Stratigraphy, sedimentology, and palaeoecology of the dinosaur-bearing Kundur section (Zeya-Bureya Basin, Amur Region, Far Eastern Russia). Geol Mag. 2005;142(6):735-750.
86. Shiki T, Kumon F, Inouchi Y, Kontani Y, Sakamoto T, Tateishi M, et al. Sedimentary features of the seismo-turbidites, Lake Biwa, Japan. Sediment Geol. 2000;135(1-4):37-50.
87. Diedrich CG. Palaeopopulations of Late Pleistocene top predators in Europe: Ice Age spotted hyenas and steppe lions in battle and competition about prey. J Paleontol. 2014;34. doi. 10.1155/2014/106203
88. Fanti F, Currie PJ, Burns ME. Taphonomy, age, and paleoecological implication of a new *Pachyrhinosaurus* (Dinosauria: Ceratopsidae) bonebed from the Upper Cretaceous (Campanian) Wapati Formation of Alberta, Canada. J Can Earth Sci. 2015;52:250-260.
89. Coard R, Dennell RW. Taphonomy of some articulated skeletal remains: transport potential in an artificial environment. J Archaeol Sci. 1995;22(3):441-448.
90. Aslan A, Behrensmeyer A. Taphonomy and time resolution of bone assemblages in a contemporary fluvial system: the East Fork River, Wyoming. PALAIOS. 1996;11(5): 411-421.
