# Supplementary material for: Over 13,000 elements from a single bonebed help elucidate disarticulation and transport of an Edmontosaurus thanatocoenosis
Source: PLoS One. 2020 May 21;15(5):e0233182. doi: 10.1371/journal.pone.0233182 (PMC7241792; doi:10.1371/journal.pone.0233182)
Supplement: Attachment — Submitted filename: Snyder et al 2020_Response to Reviewers.docx [file pone.0233182.s002.docx]

Jun Liu

Academic Editor

PlOS One

Chinese Academy of Sciences

Beijing, China

Dear Dr. Liu,

Re: PONE-D-20-01504

Please find attached a revised version of our manuscript entitled Over 13,000 elements from a single bonebed help elucidate disarticulation and transport of an Edmontosaurus thanatocoenosis*,* which we are resubmitting for further consideration in the *PLOS One*. We would like to thank you and the reviewers for your thoughtful comments, which helped us improve the manuscript during the revision process. Below, are point-by-point responses to each of the reviewers’ critiques.

Sincerely,

Jared Wood

Department of Biology
Southwestern Adventist University
100 W. Hillcrest St.

Keene, TX 76028 USA

+1 580-677-1608

j.wood@swau.edu

**Corrections Made to Manuscript**

**Reviewer 1.**

Formatting and clarity:
1. Line 152 - clarify this sentence, perhaps - Field techniques for documenting bones and their position, using . . . .

**Clarified this sentence removing ambiguity.**

2. When did the GPS system begin to be used in this research?

**Added sentence clarifying when GPS began to be used.**

3. Line 159 - should this begin a new section - perhaps Taphonomic concepts

**The sectional title is currently “Specimen Preparation and Taphonomic Classifications. We	split this title to “Specimen Collection and Preparation”, and then at the suggested break (line	159) added the title “Taphonomic Concepts and Classifications” This should make the sections	more easily understood.**

4. Line 183-186 - Perhaps move this paragraph to after line 177. It might make the logical sequence better.

**Moved this paragraph as suggested.**

5. Line 187-188 - Is this number of citizen scientists only working days at the quarry, or does it include lab work?

**Clarified the working days as those in the quarries only.**

6. Line 211-212 - should it say "much of our research", or "all of this paper"

**Changed to “this paper”**

7. Reference to Table 2 - Perhaps the first reference to Table 2 should be the one in line 314 (?)

**Eliminated first reference to Table 2 (line237). Moved Table 2 to line 317.**

8. Line 339-340 - Does this comment apply only to the Stair quarry, or to all quarries?

**Clarified that this was only Stair quarry.**

9. Line 408 - Perhaps it should be stated how the 6896 elements were chosen.

**Combining with the suggestion by Reviewer 2, we clarified how the elements were chosen,**

**adding teeth, tendons, and bone fragments.**

10. Line 490 - Could there be a brief concluding statement of this section?

**Added:**

**Thus, it appears that although there is a complex interplay of factors affecting the	disarticulation and subsequent transport of *Edmontosaurus* elements, a pattern emerges	showing a low recovery of axial and distal appendicular elements, and a high recovery of	proximal appendicular elements.**

11. Line 549-551 - Would this read more clearly if this sentence came earlier, perhaps at line 528?

**Moved and clarified.**

12. My comments on the figures are probably answered in the high resolution versions.
Fig. 2 and 3 need more clarity.

**Combining with comments by Reviewer 2, we added a line drawing for Fig. 2, and reworked**

**Fig. 3 to clarify layering and resolution.**

12. Figure 4 - Would the black dots show up better in a different color?

**Changed colors to show up better.**

13. Figure 6 - It seems that this figure would use space better if the columns were closer together, with a larger font.

**Modified as suggested.**

14. Minor points:
Line 29 - weathering, or breakage, instead of and	- **done**
Line 54 and 692 - should it be ontogenic or ontogenetic?	**- done**
Line 145 - Specimen Collection, preparation . . .	**- done**
Line 157 - Each element was also photographed . . .	- **done**

**- kept term “re-photographed”**
Line 247 - Would "are similar to" be better than "reflect"	**- done**
Line 362 - italicize the species name	- **done**
Line 504 - say "the same" rather than "this"	- **done**
Line 565 - typo in genus name	**- done**
Line 721 - clarify the phrase "skull dentaries"	**- done**

**Reviewer 2.**

1. However, the text could benefit from some significant editing and addition of citations in places, and I frequently was uncertain as to what the writing was trying to convey. A thorough pass aimed at clarification would be useful. I tried to give suggestions on potential modifications that could help when possible, which I hope the authors will consider, and pose questions in other places to note where clarification is needed. For example, there are a few linkages between taphonomic data and interpretations that sound inaccurate (e.g., lack of articulation doesn’t suggest mass mortality, but the limited ontogenetic range represented and uniform patterns of weathering and abrasion do).

**We have followed this suggestion by examining the flow of the paper, moving a few sections to where they fit best, making all the suggested changes on the returned manuscript to clarify concepts and terminology, and added several citations while eliminating one. This reviewer put a great amount of time into upgrading this manuscript, and we worked to incorporate his suggested changes.**

2. Similarly, data presented in a couple of the tables could use a little better clarification (e.g., “subtotal” lines in Table 3). Multiple figures (2, 3, 5, 6, 7, 8[component reordering]) could also use some editing to aid clarification.

**These have been modified as suggested.**

3. Scientifically, in my opinion there are five main topics that either need revision or to be addressed. First, I agree the species is most likely E. annectens, but all of the data provided in support of this conclusion are circumstantial - this assignment needs to be documented by anatomical comparisons.

**We performed additional analyses using diagnostic anatomical traits to confirm that our hadrosaur fossils are indeed *E. annectens*. These additional analyses are described in the manuscript.**

4. Second, the authors should use age categories developed for Edmontosaurus by Gangloff and Fiorillo (2010) rather than those developed for ceratopsians by Ryan et al. (2001).

**Changed to reflect aging of *Edmontosaurus* as developed by Horner (2000) instead of	ceratopsians.**

5. Third, the authors largely disregard abundant isolated teeth, ossified tendons, and bone fragments in the assemblage, but these must also be studied as their preservation states will likely provide further (and potentially very useful) insights into the transport history of the assemblage (especially given their great abundances). Presuming these are also in the collection, this addition should not require too much time.

**These have been incorporated into the data on the tables where applicable.**

6. The other two topics of importance are interconnected, and deal with the interpretation of the depositional scenario and environment. I agree the assemblage looks like a debris flow deposit, with evidence such as the normal grading of bones and paucity of lightweight skeletal elements supporting this interpretation. Because of this, it seems far more likely that the common spiral breakage observed is best attributable to relatively high energy transport of the bones in the skeletally-dense debris flow, not to postmortem trampling. Though the authors suggest that hydraulic inequivalency (sensu Behrensmeyer 1975) may reflect the remains being authochthonous, this interpretation is inconsistent with all the other data – short distance transport by a quickly moving large volume of mud can create a parautochthonous end deposit like this exhibiting hydraulic inequivalency between bones and their host matrix, and this type of event is not uncommon in fluvial environments. Finally, it appears an outdated reference (their #76) may have lead the authors partly astray in interpreting the environment of burial, specifically into considering a potential marine depositional setting for the HR Bonebed. The entire Lance Formation is now widely agreed to be of fluvial origin because the marine Cannonball Formation has been definitively clarified to be Paleocene in age, and thus it is not a member of the Lance. Many workers indeed consider the Lance to be the same deposits as the Hell Creek Formation across the MT state line, deposits of which are also of fluvial origin. Thus, the debris flow hosting the HR Bonebed must have been deposited on a floodplain. The authors’ data are consistent with this, as only terrestrial and fluvial/freshwater taxa are represented and the sedimentology of the bonebed unit and surrounding strata are consistent with fluvial channel sands and floodplain mudstones. Though the comments in my review on this point are long, it is really a rather minor point that impacts only where you interpret the final site of burial to be. The primary changes will mostly be altering phrases from “marine” or “deltaic” to “aquatic” or “fluvial”, and so forth.

**Removed outdated reference #76 and the text referring to it. Incorporated much more recent	references and clarified the possible interpretations of this bonebed with the suggested**

**terminology.**

**Editor comments.**

1. Please ensure that your manuscript meets PLOS ONE's style requirements, including those for file naming. The PLOS ONEstyle templates can be found at

**We have done our best to make sure our paper and figures are formatted according to PlosOne guidelines.**

2. We note that Figures 1 and 4 in your submission contain map and satellite images which may be copyrighted.

**Figures 1 and 4 were created by authors and did not use outside sources. Thus copyrighting is not an issue.**
